# Genetic characterisation of wild ungulates: successful isolation and analysis of DNA from widely available bones can be cheap, fast and easy

**DOI:** 10.3897/zookeys.965.54862

**Published:** 2020-09-03

**Authors:** Elena Buzan, Sandra Potušek, Felicita Urzi, Boštjan Pokorny, Nikica Šprem

**Affiliations:** 1 University of Primorska, Faculty of Mathematics, Natural Sciences and Information Technologies, Glagoljaška 8, 6000, Koper, Slovenia University of Primorska Koper Slovenia; 2 Environmental Protection College, Trg mladosti 7, 3320, Velenje, Slovenia Environmental Protection College Velenje Slovenia; 3 Slovenian Forestry Institute, Večna pot 2, 1000, Ljubljana, Slovenia University of Zagreb Zagreb Slovenia; 4 Department of Fisheries, Apiculture, Wildlife Management and Special Zoology, University of Zagreb, Faculty of Agriculture, Svetošimunska cesta 25, 10000, Zagreb, Croatia University of Zagreb Zagreb Croatia

**Keywords:** amplification success, bones, demineralisation, DNA extraction, population genetics, skulls, wild ungulates

## Abstract

Genetic characterisation of wild ungulates can be a useful tool in wildlife management and in obtaining a greater understanding of their biological and ecological roles in a wider spatiotemporal context. Different ways of optimising methodologies and reducing the costs of genetic analyses using widely available bone tissues collected within regular hunting allocations were examined. Successful isolation and analysis of DNA from widely available bones can be cheap, fast and easy. In particular, this study explored the possibility of using bones for extracting high quality nuclear DNA for microsatellite analysis. The utility of applying a modified demineralisation process using two commercially available DNA isolation kits, which differ significantly in price, was evaluated. The sample sets included bones and, for comparison, muscle tissues from four wild ungulate species: chamois (*Rupicapra rupicapra*), roe deer (*Capreolus capreolus*), wild boar (*Sus scrofa*), and Alpine ibex (*Capra ibex*). For the recent bones, these results confirmed that the DNA concentrations and microsatellite amplification were sufficiently high, even when using low-cost kits, after prior demineralisation. For old bones, prior demineralisation and use of a specially designed isolation kit led to a more successful extraction of DNA. Besides reducing kit-related costs, low-cost kits are much faster and therefore make genetic analysis more efficient.

## Introduction

Genetic and genomic approaches can provide detailed information about past and present demographic parameters in populations, phylogenetic issues, the molecular basis for understanding genetic diseases, inbreeding, and detection of hybridisation/gene introgression (Miller et al. 2012). Genetic monitoring can also help us to understand the mechanisms that relate fitness to genetic variation, to integrate genetic and environmental methodologies into wildlife management and conservation biology, and to design advanced and fast (as well as non-invasive) monitoring protocols. Indeed, the rapid progress and decreasing costs of genetic analyses are making genetic monitoring more feasible and more widely applicable (Ouborg et al. 2010).

Nevertheless, the collection of genetic material from free-ranging animals is very challenging. At present, the ability to perform routine, large-scale genetic analyses and monitoring in a wider spatiotemporal context is quite low due to the limited availability of suitably preserved soft tissue samples (e.g. muscle tissue or internal organs) from wildlife species. The difficulty in collecting genetic material from wildlife is further exacerbated because traditional invasive techniques such as biopsy darting or blood sampling (the preferred biomaterial for genetic studies) are impractical and need appropriate preservation methods (Seutin et al. 1991). Moreover, it is often difficult to acquire a large enough set of historical samples, such as different bone structures, which are also more difficult and expensive to analyse (Hoffmann et al. 2015).

To encourage the use of genetics as a valuable management tool, we should optimise the methodology and reduce the costs of genetic analyses on widely available samples, both recent and historical, that are systematically collected by hunters as trophies (such as skulls with antlers or horns) or, in some cases, via their reporting obligations (mandibles). Indeed, in some European countries such as Slovenia, (hemi)mandibles of all harvested wild ungulates must be collected, properly labelled, linked with attributive data on the individual, and provided as part of regular hunting allocations (Pokorny and Jelenko Turinek 2018). Therefore, these mandibles represent a very useful resource and an easily accessible set of samples for both regular and genetic monitoring, such as for studying patterns in the genetic structure of species across the landscape, their hybridisation patterns and fundamental life-history traits. The same holds true for trophies, which also yield data within a given spatiotemporal context (i.e. information on the location and date of the harvest). Using regularly collected bone samples helps us to avoid the rather complex logistics involved in the sampling and storage of muscle tissue samples.

Bone structures that are equipped with attributive data on individuals enable us to study the spatiotemporal variability within and among populations and to perform retrospective studies (e.g. mandibles in different environmental studies: Kierdorf and Kierdorf 2000, 2005; Jelenko and Pokorny 2010; antlers/horns in genetic studies: Hoffmann and Griebeler 2013; Giżejewska et al. 2016; Safner et al. 2020). These bones are usually kept for decades or even centuries in well-preserved public (museums) and private collections, which may cover different regions and historical periods, and in which data on the origin of individuals, year of death and age are also often recorded.

However, bone tissue is one of the most difficult biological samples for the extraction of DNA (Wandeler et al. 2007), and working with such material is both time- and labour-consuming (Alonso et al. 2001). Luckily, improvements in the extraction of quality DNA (which used to be a limiting factor) and a significant reduction in costs related to DNA analyses of bones (Paetkau 2003; Rohland and Hofreiter 2007; Dabney et al. 2013; Huisman et al. 2016; Tsaparis et al. 2019) have opened up completely new perspectives for genetics as a wildlife management tool. This is primarily because bones, due to their structure, preserve DNA comparatively well and for a long time. Therefore, they are a useful source of genetic material but, compared with extracting DNA from muscle tissues, hair and saliva, are more difficult to handle due to their rigid structure (Kumar and Narayan 2014).

The quality and quantity of the DNA extracted from bones can be affected by several factors such as the mineralisation levels, the pre-treatment of the bone prior to the extraction step and the environmental conditions under which it was preserved. The extraction process in bone also presents two big problems: low copy number due to the fact that the majority of bone is not cells (Loreille et al. 2007), and the occurrence of incomplete or broken DNA fragments and contamination by PCR inhibitors (Chilvers et al. 2008). Therefore, isolating and purifying DNA from bones, particularly old ones, pose a huge challenge (Rohland and Hofreiter 2007; Dabney et al. 2013). The bone tissue itself is a hard, connective tissue with a high content of calcium. After death, during mineralisation and protein decay, DNA is bound with hydroxyapatite and forms a compound named bioapatite (Dorozhkin 2016). For these reasons, decalcification (softening the bones) is generally considered a necessary step for effective DNA isolation (Alers et al. 1999). However, we should take into consideration the influence of the decalcification period in old bones: Jakubowska et al. (2012) showed that for well-preserved bones decalcification length does not play an important role, while, on the contrary, degraded older bones should be decalcified for a couple of days to obtain better results from DNA isolation.

The aims of our study were to: (i) determine a low-cost and effective method for extracting high quality DNA from mandibles and skulls of harvested wild ungulates, using chemicals and tools readily available in the majority of molecular laboratories; (ii) compare the success rate of DNA extraction and amplification from bones (both recently collected and old ones) *vs.* muscle tissue samples using low-cost (wider use for all types of tissue) and high-cost isolation kits (specially made for DNA extraction from bones); (iii) rate genotyping reproducibility by genotyping seven roe deer samples shed by the same individual.

## Materials and methods

### Sampling and samples

We collected samples of four wild ungulate species: chamois (*Rupicapra rupicapra*), roe deer (*Capreolus capreolus*), wild boar (*Sus scrofa*), and Alpine ibex (*Capra ibex*) (Table 1). For each species (except ibex for which muscle tissue samples were not at our disposal) we used muscle tissue and recent bone (from animals hunted in 2017), as well as old bone (from 20- to 100-years-old) samples.

**Figure 1.**
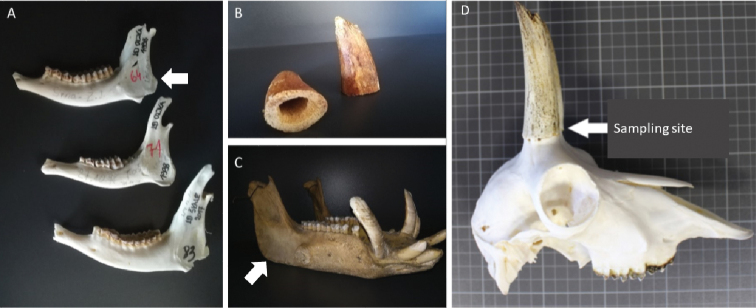
Micro-locations of sampling bone material for DNA isolation (see arrows) **A** roe deer mandibles **B** ethmoid bone of chamois **C** wild boar mandible **D** chamois skull.

Bone samples comprised hunting trophies (skulls with antlers/horns) or left hemi-mandibles and were provided by the Slovenian and Croatian hunting authorities. Considering the importance that trophies have for both owners and historical records, we were careful not to destroy their physical appearance and value. Therefore, in the case of trophies, we either sampled ethmoid bone (i.e. an exceedingly light and spongy bone that separates the nasal cavity from the brain) or the cornual process (a conical bony core which is fully overgrown by horn) (Figure 1).

All recently culled individuals of each species were hunted during regular hunting activities according to relevant yearly hunting management plans and the national legislation of both countries. We used only samples from already dead individuals, therefore no animal was shot or killed by any other means for the purposes of this research.

For microsatellite genotyping, we used sample set of 67 recent bones, 40 old bones, and 493 muscle tissues. For determining the amplification success by qPCR, we pooled them into a subset of 50 recent bones, 40 old bones, and 18 muscle tissue samples (Table 1).

**Table 1.** Summary data on samples investigated during the study (n – number of samples used for the subset in qPCR assay).

Sample ID	Species	*n*	Sample type	Organ/tissue	Year of harvest	Origin of the animal
RR1	Chamois	6	tissue	muscle	2017	Croatia
RR2		12	recent bone	skull (ethmoid bone)	2017	Croatia
RR3		13	old bone	skull (cornual process)	>80 years old	Croatia
CC1	Roe deer	7	tissue	muscle	2017	Slovenia
CC2		19	recent bone	mandible	2017	Slovenia
CC3		16	old bone	mandible	>20 years old	Slovenia
SS1	Wild boar	5	tissue	muscle	2017	Slovenia
SS2		13	recent bone	mandible	2017	Slovenia
SS3		7	old bone	mandible	>100 years old	Croatia
CI1	Alpine ibex	6	recent bone	skull (ethmoid bone)	2017	Slovenia
CI2		4	old bone	skull (ethmoid bone)	>30 years old	Slovenia

### Contamination prevention in old bones

To avoid possible cross-contamination, laboratory work with old bone samples was conducted in a dedicated, physically-isolated laboratory for ancient DNA. Strict protocols for personnel hygiene were followed to minimise the amount of human DNA. In addition, personnel movements from the post-PCR environment to the extraction DNA laboratory were avoided to prevent the flow of atmospheric DNA. All surfaces in the lab were routinely double wiped with bleach and rinsed with absolute ethanol. After each analytical event, the laboratory was irradiated with ultraviolet light for at least two hours. All consumables, disposables, tools, and instruments were externally bleached and UV irradiated before entering the lab, as well as being subjected to routine cleaning before, during and after use.

### DNA extraction protocol

Recent and old bone samples were carefully shredded using a sterilised sanding tool and scalpel blade. Before isolation, we proceeded with the following demineralisation protocol to obtain as much DNA as possible. Extraction from recent and old bone samples was performed using four combinations of two kits and with/without demineralisation procedures as follows: (i) PeqLab with demineralisation; (ii) PeqLab without demineralisation; (iii) Qiagen with demineralisation; and (iv) Qiagen without demineralisation.

We weighed approximately 0.3 g (± 0.05 g) of bone powder into a sterile 50 ml falcon tube, added 10 ml of 0.5 M Ethylenediaminetetraacetic acid (EDTA) and vortexed thoroughly. We incubated overnight at 37 °C with gentle agitation. Another 50 ml falcon tube was used for isolation of the blind control, which consisted of the same set of reagents but without bone powder.

We then centrifuged samples at 1300×g for 15 min; a pellet of residual powder was typically seen at this point. The supernatant was discarded. In the extraction for the negative control, we left approximately 100 μL of supernatant. We added 10 ml of sterile bi-distilled water and vortexed at high speed for 10 sec, and centrifuged again at 1300×g for 15 min.

After demineralisation of the samples, we proceeded with isolation of DNA by adding 100 µL of lysis buffer (ATL buffer -Qiagen or Lysis buffer T -PeqLab), 60 µL of Proteinase K and 20 µL of Dithiothreitol (DTT) to the pellet, and then we incubated the samples at 56 °C for 2–3 hrs. After this step, we followed an isolation protocol using the QIAamp DNA Micro Kit (Qiagen; thereafter: QIAamp) or Tissue DNA Mini Kit–S line (VWR International, Leuven, Belgium; thereafter: PeqLab), according to the manufactures’ instructions. DNA was eluted in the respective kit elution buffer, then sample concentrations were normalised and dilutions were stored in a refrigerator at 4 °C to reduce thaw-freeze cycles.

We used muscle tissue as a control for each studied species (except Alpine ibex), since muscle is one of the preferred sample types for genetic studies: 2×2 mm of tissue samples, air-dried under sterile conditions in order to remove the ethanol, were used for DNA extraction using the Tissue DNA Mini Kit–S line (VWR International, Leuven, Belgium; PeqLab), according to manufacturer’s instructions.

### DNA concentration and quantification

The concentration and purity of the obtained DNA in the final elution volume was measured using a Qubit dsDNA BR Assay Kit (Invitrogen) on a 3.0 Qubit Fluorimeter (Life Technologies). Additionally, the spectral curve was measured on an Epoch Microplate Spectrophotometer (BioTeck) using Gene5 v.1 software to check for potential impurities. We expressed the obtained amount of DNA in ng per µL of DNA in final elution volume. The quality of DNA obtained from samples was assessed using Sybergreen chemistry. All qPCR reactions were performed on a Roche LightCycler 96 System (Roche Diagnostic GmbH, Germany) under the conditions described in Table 1 (Suppl. material 1). Degradation of DNA samples was determined by calculating the amplification success of the 100 bp and 200 bp products given by quantification cycle value (Cq value), which were generated directly at a specific threshold. The MIQE guidelines (Bustin et al. 2009) suggested that Cq < 40 has to be set as a threshold for positive amplification. The Cq is defined as the number of cycles needed for the fluorescence signal to reach a specific threshold. A predefined threshold is set within the exponential amplification phase, when doubling of the product can be detected above background fluorescence, and the number of cycles needed to reach this threshold is used to estimate the amount of template DNA present. By comparing the Cq values between two samples we can compare the amount of DNA fragments in one sample relative to the other. Lower Cq values mean higher initial copy numbers of the target.

We selected the fragment lengths of 100 bp and 200 bp according to: (i) the recommendation for qPCR that optimal amplicon length should be less than 200 bp (Nielsen et al. 2017); (ii) the fact that the majority of selected microsatellite alleles were around 200 bp or less in length. For quantification, all samples were analysed in triplicate. Negative controls were included alongside every batch of samples processed. The blank sample did not produce any measurable amplicons.

### Microsatellite analysis

The multilocus genotyping of microsatellites was used: (i) to quantify the success rate of amplification using a larger sample set (see Table 2), and (ii) to evaluate the reproducibility of genotyping using recent bone and muscle tissue from the same roe deer. The second analysis was possible because bone and tissue samples from the same individual were available. In this individual, we performed a microsatellite analysis in seven DNA isolation replicates to confirm the multilocus genotype, and to estimate the possible proportion of mismatches between the replicated analyses.

**Table 2.** Overview of the results of different DNA extraction methods used for different samples. Number of samples (n) used in qPCR assays is summarized for all species. Average of DNA yield (concentration) achieved with different methods (means ± SD). DNA quality for different extraction methods/samples is given by quantification cycle scores Cq100, Cq200 and as ratio Cq100/Cq200 (means ± SD), which also indicates DNA degradation. The amplification success is given in percentages for Cq200 values. The microsatellite genotyping for muscle tissue, recent bones (PeqLab D and WD) and old bones (QIAamp D) success is calculated on larger samples sets (67 recent bones, 40 old bones, and 493 muscle tissues, respectively).

Sample	Isolation method	*n*	Concentration (ng/μL)	Cq100	Cq200	Ratio *(Cq100/Cq200)*	Amplification success Cq200 (%)	*p* – value (χ*^2^*- posthoc test)	Microsatellite success rate (%)
Muscle tissues	PeqLab	18	36.30 ± 13.91	26.51 ± 6.27	25.15 ± 4.32	1.04 ± 0.19	94.7	0.19	96%
Recent bones	PeqLab WD	18	25.43 ± 26.89	28.54 ± 2.71	31.30 ± 2.56	0.79 ± 0.05	61.1	**0.00**	90%
Recent bones	PeqLab D	12	45.31 ± 20.59	28.01 ± 2.17	37.03 ± 1.19	0.75 ± 0.05	91.7	0.84	97%
Recent bones	QIAamp WD	8	16.15 ± 19.05	27.80 ± 1.22	36.08 ± 2.52	0.77 ± 0.03	87.5	0.48	89%
Recent bones	QIAamp D	12	25.29 ± 15.39	24.29 ± 2.46	26.94 ± 1.41	0.90 ± 0.11	100	0.11	95%
Old bones	PeqLab WD	8	1.90 ± 1.69	29.44 ± 3.41	35.56 ± 0.94	0.80 ± 0.10	25.0	**0.01**	60%
Old bones	PeqLab D	8	17.04 ± 19.84	28.81 ± 7.56	37.45 ± 5.08	0.74 ± 0.17	37.5	0.09	72%
Old bones	QIAamp WD	12	1.05 ± 0.76	27.27 ± 5.58	35.25 ± 4.03	0.74 ± 0.09	58.3	0.62	68%
Old bones	QIAamp D	12	5.11 ± 5.61	26.79 ± 3.31	31.22 ± 5.72	0.93 ± 0.13	66.7	0.84	78%

Notes: WD = without demineralisation, D = with demineralisation. To determine differences between individual pairs of variables (amplification success) for χ^2^-posthoc tests we used Bonferroni correction due to multiple comparisons, therefore p-value of statistical significance was set as *p* < 0.01.

### PCR reaction and genotyping of microsatellites

The DNA extracted from bones and muscle tissue was treated identically. Microsatellites were amplified using Alltaq PCR Core kit (Qiagen), using 3 µL of template DNA under the following conditions: initial PCR activation for 2 min at 95 °C followed by 40 cycles with denaturation for 10 s at 95 °C, annealing for 30 s at 55 °C, extension for 20 sec at 72 °C, and final extension for 10 min at 72 °C. Fragment analysis was performed on a SeqStudio sequencer (Thermofischer scientific) using a GeneScan LIZ500 (-250) standard (Applied Biosystems). Results were validated using GeneMapper v.5.0 software (Applied Biosystems).

To quantify the amplification success rate, all samples were genotyped at three microsatellite loci (approximately 100 bp and 200 bp long) with species-specific microsatellites primers as follows: roe deer (Kuehn et al. 2007), chamois and Alpine ibex (Zemanová et al. 2011), and wild boar (Robic et al. 1995) (Suppl. material 1: Table S2).

To rate genotyping reproducibility, we amplified 14 microsatellite loci in 4 multiplex sets, containing 4, 2, 3 and 4 microsatellites, although locus MAF70 was carried out separately using the protocol described in Kuehn et al. (2007), which is commonly applied in population genetic studies utilising DNA from the muscle tissue of roe deer. Following the same procedure, we amplified 20 microsatellites in 4 multiplex sets (Zemanová et al. 2011) for chamois bones samples (Suppl. material 1: Tables S3, S4). We compared microsatellite amplifications of DNA extracted from recent bones and muscle tissue by genotyping seven samples shed by the same individual (roe deer).

### Statistical analyses

All statistical analyses were performed using SPSS software Version 25 (SPSS Inc., Chicago IL). We used the Shapiro-Wilk’s test to check the normality of: (i) DNA concentrations (yield), and (ii) quantification cycle data: Cq100 and Cq200 values. Since neither set of data was normally distributed, we used nonparametric statistics in all subsequent analyses. In the following sections, all continuous data are presented with means and standard deviations, while categorical data are presented as numbers and percentages.

The differences in DNA yields were assessed by comparing measured concentrations of DNA isolated from muscle tissue *vs.* recent bones *vs.* old bones by different isolation methodologies. We used the Kruskal-Wallis test for testing the significance of differences in DNA yield between different isolation methodologies. The effect of the demineralisation protocol on bones’ DNA yield was assessed by the Mann-Whitney U test.

We assessed the quality of isolated DNA by comparing the quantification cycle data: Cq values measured for 100 bp (short) and 200 bp (long) fragments in qPCR assays. The DNA quality was assessed by: (i) the ratio between Cq100/Cq200 value as was described previously by Nielsen et al. (2017), and (ii) the amplification success expressed in percentages for Cq200, which correlate with a sufficient amount of 200 bp DNA product to be detected.

For testing the significance of differences in the ratio Cq100/Cq200, we used the Kruskal-Wallis test. The *χ^2^*-test was used to compare Cq200 amplification success for all isolation methods, where we set the success threshold of Cq < 40 as described in the MIQE guidelines (Bustin et al. 2009), and then coded with “1” each result with Cq < 40 as successfully amplified, and unsuccessfully amplified PCR with the code “0” (Cq > 40).

To assess the pairwise comparison of Cq200 amplification success we used the *χ^2^*-posthoc test for adjusted residuals with a Bonferroni correction due to multiple comparisons.

All statistical analyses for recent and old bones were performed separately, and the level of statistical significance was set as *p* < 0.01 in all analyses.

## Results

### DNA yield

All extraction methods used resulted in different DNA concentrations (between 0.23 ng/µL and 82.4 ng/µL; mean = 16.4 ± 20.0 ng/µL). Regardless of the species, the amounts of DNA extracted from muscle tissues were 18.8–52.7 ng/µL (mean 36.3 ± 13.9 ng/µL), from recent bones 1.40–82.4 ng/µL (mean 27.1 ± 21.9 ng/µL), and from old bones 0.23–50.3 ng/µL (mean 5.41 ± 10.7 ng/µL), respectively. DNA concentration in the extracted blank sample was not detected (see Table 2).

There were no statistical differences in recent bones’ DNA concentrations isolated by different methods in comparison to tissue DNA concentration (*H* = 9.028; *p* = 0.060), but we did find significant differences when samples were prepared either with demineralisation or when this step was excluded (*H* = 11.916; *p* = 0.008). Interestingly, the methodology that includes demineralisation and the low-cost PeqLab kit isolation gave the highest DNA yield (45.3 ± 20.6 ng/µL), but DNA isolation without prior preparation of samples led to significantly lower DNA concentrations (*H* = 25.048; *p* = 0.010).

On the other hand, we found significant differences among old bones’ DNA concentrations when comparing different isolation methodologies (*H* = 22.938; *p* < 0.001). There was a clear tendency for old bone samples to yield significantly less DNA compared with either recent bones (*H* = 30.141; *p* < 0.001) or tissue samples (*H* = 38.797; *p* < 0.001). The highest and significant differences (*H* = 28.900; *p* < 0.001) in DNA concentrations between old bones and muscle tissues were found when using the QIAamp kit isolation without demineralisation. However, this DNA concentration was significantly lower than the concentrations obtained either with the PeqLab kit with previous demineralisation (*H* = 15.004; *p* = 0.025) or with QIAamp kit with previous demineralisation (*H* = 12.976; *p* = 0.038).

### DNA quality

#### Recent bones

All samples were amplified successfully using qPCR. Cycle quantification Cq100 scores for recent bones were comparable between different methods, but slightly lower for the QIAamp kit isolation without demineralisation (24.29 ± 2.46). The same pattern was also observed for Cq200 (26.94 ± 1.41). The Cq100/Cq200 ratios were above 70% for all recent bone samples, with the highest value observed for QIAamp isolation with demineralisation (0.90 ± 0.11).

Short fragments (Cq100) were successfully amplified regardless of the method used (χ^2^-posthoc test: *p* = 0.482). The amplification success for Cq200 differed between methods (*p* = 0.015). The proportion of successfully amplificated Cq200 fragments for recent bones using a demineralisation protocol or muscle tissues was > 90%. Significantly, the least successful Cq200 amplification was found in DNA fragments isolated with the PeqLab kit without demineralisation (61.1%; *p* < 0.001).

#### Old bones

Cycle quantification scores for short fragments (Cq100) were comparable between all methods used (*p* = 0.103). However, the amplification success of long fragments differed between methods (*p* = 0.003), and was, again, significantly lower for PeqLab isolation without demineralisation (*p* < 0.001). The proportion of successfully amplificated Cq200 fragments was only 25.0% for the PeqLab kit without demineralisation and 37.5% for the PeqLab kit with demineralisation. The proportion of successfully amplificated Cq200 fragments was higher for the QIAamp kit: 58.3% without demineralisation and 66.7% with the demineralisation protocol. Short fragments amplified more effectively due to the lower concentration of longer fragments in degraded DNA from old bones.

### Microsatellites

Microsatellite analysis showed that muscle tissue samples and recent bones isolated with and without demineralisation protocol (using both kits) produced amplicons with the correct microsatellite loci positions (see Suppl. material 2) for short (80–100 bp), medium (approximately 150 bp), and longer fragments (200–250 bp). Amplification produced identical fragment lengths per locus (80–300 bp), similar allele numbers, as well as comparable signal strength and allele scoring. The amplification success rate for microsatellite genotyping is shown in Table 2, which provides an overview of the results of the different DNA extraction methods used for different samples.

Quality parameters such as the height of peaks and the presence of non-specific peaks were comparable between the two kits, with fewer non-specific peaks observed when using demineralisation protocols. For old bones, the highest microsatellite success (> 78%) and higher-quality parameters were observed for DNA isolated using the QIAamp kit with prior demineralisation. However, the amplification of DNA samples was more successful when the demineralisation protocol was used (> 95%). Without prior preparation of the bones, the amplification success decreased to 90%, and in some cases longer alleles were not amplified in the multiplex (in 15% of the analyses).

The replicated DNA of both muscle tissue and bone samples of the same roe deer individual showed the same multilocus genotype.

## Discussion

Despite many improvements, genetic analysis has not seen widespread use as a tool in wildlife management, in part due to the perception that genetic analyses are expensive and that sample collection is difficult (Holderegger et al. 2019). This study shows how to reduce the costs of genetic analyses on widely available samples, i.e. bony structures that are systematically collected by hunters as trophies (skulls with antlers or horns) or under reporting obligations (mandibles). Such bony structures are very favourable for genetic analyses due to the high level of DNA preservation as a result of careful storage and pre-treatment of the material immediately after harvesting. Thus, when an animal is culled, the head is removed immediately and cooked to remove all soft tissues from the cranium. The dry and constant environmental conditions in which trophies and mandibles are usually stored are ideal for the preservation of high-quality DNA over a longer timescale.

The recent bones used in this study showed DNA content and DNA amplification success rates equal to that of muscle tissue samples. DNA concentrations obtained using our low-cost extraction methods were sufficient for microsatellite analysis (Table 2, Suppl. material 2). There was no evidence for a scoring error due to stuttering, allelic dropouts or null alleles for any of the microsatellite loci for the recent bones’ DNA. In the case of old bones, DNA isolated with more expensive kits (specially designed for bones) and using a demineralisation protocol showed fewer genotype errors and better amplification success.

The finding that the DNA isolated from recent bones using low-cost methods showed amplification success rates equal to DNA isolated from muscle tissue is astounding. We also revealed that a modified isolation protocol, i.e. using the kit after prior demineralisation of samples instead of the classical phenol/chloroform isolation method, reduced the time needed for the procedure by at least twofold and that costs could be reduced by at least 50% due to the possibility of a using widely available low-cost kit.

We have demonstrated that bones, even old ones (> 20 years old), are a good source of DNA even if they have been previously treated with aggressive agents (i.e. hydrogen peroxide), as was the case for almost all of our samples. Where such samples and sample sets are available, even for large-scale studies in different spatiotemporal contexts, there is no need to collect additional soft tissues or non-invasive samples for genetic studies. Indeed, both low- and high-cost commercial kits showed high recovery of DNA after previous bone demineralisation (all concentrations were much higher than 16 ng/μL), even in the case of very old bones (> 100 years old). Past studies have shown the importance of developing and optimising DNA isolation methods from analytically difficult samples such as bones (Loreille et al. 2007; Rohland and Hofreiter 2007; Seo et al. 2009; Nielsen et al. 2017). For example, Loreille et al. (2007) proposed a complete demineralisation protocol, which results in full physical dissolution of the bone samples, to maximise DNA yield.

It has been previously suggested that geneticists should always use demineralisation of bones for successful DNA isolation (Jakubowska et al. 2012). However, we have shown that high DNA yields can be recovered from well-preserved bone samples even without demineralisation, presumably because free DNA was not washed away. According to our results, well-preserved bones do not need to undergo a demineralisation process, which adds more handling and pipetting steps, thereby increasing the extraction time and costs, and also increasing the possibility of secondary contamination and errors. Nevertheless, we have to stress that the success rate was also better in recent bones when using demineralisation pre-treatment of samples.

A high variability in DNA concentrations in the subset of recent bones could be a consequence of differences in bones density (Pinhasi et al. 2015), interspecies variability, the sampling micro-location at the skull (especially when using antlers or the cornual process in horns), different distribution and variable post-mortem preservation of DNA across/within individual bone. However, in spite of this variability, the lower extracted concentrations of DNA were sufficiently high for genotyping and were comparable to the concentrations of DNA isolated from muscle tissues. It is very important for successful genotyping that the extracted DNA contain an adequate quantity of longer fragments (> 250 bp), particularly in recent bone samples.

In the case of old bones, DNA concentrations varied even more, probably due to the low quantity of endogenous DNA caused by poor preservation and the high likelihood of DNA degradation. Diagenesis has variable effects in old bones and can cause localised differences in bone structure, for example, the bone is less compact and more porous at sites of muscle attachment than in the surrounding bone (Hawkey and Merbs 1995; Mann et al. 2013). Therefore, this site may be more susceptible to diagenetic processes, which increase as bones age. Differences in diageneses processes influence the variation in sampling (Rohland and Hofreiter 2007) and, consequently, DNA concentration. The demineralisation process increases DNA yield, because EDTA demineralises the bones and inactivates DNA by chelating bivalent cations such as magnesium and calcium ions (Loreille et al. 2007). In our study, the demineralisation process significantly increased the DNA yield for both isolation kits, but only the high-cost commercial kit showed adequate amplification success for long fragments (Cq200), while the low-cost kit was adequate for microsatellite genotyping. Nevertheless, our results revealed that even when using (very) old bones, using a demineralisation process allows for more successful extraction of DNA.

## Conclusion

Our findings suggest that genetic population studies can be easily conducted using well-preserved bones even without any prior preparation of the bones for DNA isolation. This means that genetic studies, such as genetic identification of individuals or paternity analyses, can be carried out using readily available bony structures (trophies and particularly mandibles) in an easy, fast and inexpensive way. Moreover, the fact that mandibles are collected for both sexes and for both juveniles and adults allows the acquisition of large sample sets without sex- or demographically-biased data. This approach enables further genetic insights into various ecological and management issues for all wild ungulates for which such bony structures are available, whether on the national, regional or larger scale.

## Supplementary materials

Supplementary material 1 Tables S1–S4 Data type Microsoft Word 97 - 2003 Document (.doc) Explanation note **Table S1.** Conditions in qPCR assays. **Table S2.** Selected microsatellite loci amplified in qPCR assays or microsatellite analysis. **Table S3.** Microsatellite loci analyzed in fragmentation analysis of *R. rupicapra*. **Table S4.** Microsatellite loci analyzed in fragmentation analysis of *C. capreolus*. https://binary.pensoft.net/file/449235This dataset is made available under the Open Database License (http://opendatacommons.org/licenses/odbl/1.0/). The Open Database License (ODbL) is a license agreement intended to allow users to freely share, modify, and use this Dataset while maintaining this same freedom for others, provided that the original source and author(s) are credited.Elena Buzan, Sandra Potušek, Felicita Urzi, Boštjan Pokorny, Nikica Šprem

Supplementary material 2 Microsatellite loci for muscle tissue samples and recent bone samples Data type Microsoft Word 97 - 2003 Document (.doc) Explanation note **Figure S1.** Microsatellite loci for muscle tissue samples (upper) and recent bone samples (bottom) of *R. rupicapra* isolated using the cost-efficient PeqLab kit. **Figure S2.** Microsatellite loci for muscle tissue samples (bottom) and recent bone samples (upper) of *C. capreolus* isolated with cost efficient PeqLab kit. https://binary.pensoft.net/file/449236This dataset is made available under the Open Database License (http://opendatacommons.org/licenses/odbl/1.0/). The Open Database License (ODbL) is a license agreement intended to allow users to freely share, modify, and use this Dataset while maintaining this same freedom for others, provided that the original source and author(s) are credited.Elena Buzan, Sandra Potušek, Felicita Urzi, Boštjan Pokorny, Nikica Šprem
