# Supplementary material for: Genetic characterisation of wild ungulates: successful isolation and analysis of DNA from widely available bones can be cheap, fast and easy
Source: Zookeys. 2020 Sep 3;965:141–56. doi: 10.3897/zookeys.965.54862 (PMC7483325; doi:10.3897/zookeys.965.54862)
Supplement: Supplementary material 1 — Tables S1–S4 [file zookeys-965-141-s001.doc]

**Supplementary Material 1**

**Table 1:** Conditions in qPCR assays

|  |  | Cycles | T (°C) | Time (s) | Ramp  (°C/s) |
| --- | --- | --- | --- | --- | --- |
| Amplification  Melting reaction | Preincubation | 1 | 95 | 600 | 4.4 |
|  | Denaturation | 45 | 95 | 10 | 4.4 |
|  | Annealing |  | 60-(0.5)/cycle | 10 | 2.2 |
|  | Elongation |  | 72 | 10 | 4.4 |
|  | Denaturation | 1 | 95 | 10 | 4.4 |
|  | Annealing |  | 65 | 60 | 2.2 |
|  | Elongation |  | 97 | 1 | / |

**Table 2:** Selected microsatellite loci amplified in qPCR assays or microsatellite analysis

| Primer pairs  used | Sequence 5'–3' | Species | | Fragment length |
| --- | --- | --- | --- | --- |
| **OarFCB20*** | GGAAAACCCCCATATATACCTATAC | *R. rupicapra* | | 80–102 bp |
|  | AAATGTGTTTAAGATTCCATACATGTG |  |  |  |
| **ETH225** | GATCACCTTGCCACTATTTCCT |  |  | 128–150 bp |
|  | ACATGACAGCCAAGCTGCTACT |  |  |  |
| **ETH10**** | GTTCAGGACTGGCCCTGCTAACA |  |  | 200–208 bp |
|  | CCTCCAGCCCACTTTCTCTTCTC |  |  |  |
| **Roe8*** | AAGCCGCGCTTGAAGGAG | *C. capreolus* | | 80–100 bp |
|  | ATCAAGCTCCCCTCTTCG |  |  |  |
| **ETH225** | GATCACCTTGCCACTATTTCCT |  | 128–150 bp | |
|  | ACATGACAGCCAAGCTGCTACT |  |  |  |
| **BM1818**** | AGCTGGGAATATAACCAAAGG |  | | 200–250 bp |
|  | AGTGCTTTCAAGGTCCATGC |  |  |  |
| **So26*** | AACCTTCCCTTCCCAATCAC | *S. scrofa* | | 100 bp |
|  | CACAGACTGCTTTTTACTCC |  |  |  |
| **S355**** | TCTGGCTCCTACACTCCTTCTTGATG |  |  | 200–220 bp |
|  | TTGGGTGGGTGCTGAAAAATAGGA |  |  |  |
| **OarFCB304*** | CCCTAGGAGCTTTCAATAAAGAATCGG | *C. ibex* | | 100 bp |
|  | CGCTGCTGTCAACTGGGTCAGGG |  |  |  |
| **BM1258**** | GTATGTATTTTTCCCACCCTGC  ATCAAGCTCCCCTCTTCG |  |  | 200 bp |

Short (100 bp)* and long (200 bp)** fragments were used in the qPCR reaction in a LightCycler 96 (Roche). All microsatellite loci described above were also analyzed on the SeqStudio Sequencer (ThermoFischer Scientific).

**Table 3:** Microsatellite loci analyzed in fragmentation analysis of *R. rupicapra*

| PCR multiplex | Used primer pairs | Sequence5'–3' | Fluorescent labeling | Fragment length |
| --- | --- | --- | --- | --- |
| **SET1** | **OarFCB20** | GGAAAACCCCCATATATACCTATAC | PET | 80–104 bp |
|  |  | AAATGTGTTTAAGATTCCATACATGTG |  |  |
|  | **OarFCB304** | TGGAAACAATGTAAACCTGGG | FAM | 126–146 bp |
|  |  | CCCTAGGAGCTTTCAATAAAGAATCGG |  |  |
|  | **SRCRSP5** | CGCTGCTGTCAACTGGGTCAGGG | VIC | 154–174 bp |
|  |  | GGACTCTACCAACTGAGCTACAAG |  |  |
|  | **SY84** | GAACTGAACTTGTTAGTATGTTGGG | NED | 170–185 bp |
|  |  | TTGTTATGCTTGATGTTATTTTGTTAC |  |  |
|  | **SY58** | CTATTGAACCTGTATCTCCCCC- | PET | 199–212 bp |
|  |  | GCATTCTGGCTCTGGCAA |  |  |
|  | **CSSM66** | ACACAAATCCTTTCTGCCAGCTGA | FAM | 193–255 bp |
|  |  | AATTTAATGCACTGAGGAGCTTGG |  |  |
|  | **ETH10** | GTTCAGGACTGGCCCTGCTAACA | NED | 205–217 bp |
|  |  | A CCTCCAGCCCACTTTCTCTTCTC |  |  |
| **SET2** | **SY434** | AAGTGTCTGGGTTCTCTTTCTCTA | NED | 77–103 bp |
|  |  | ATGTCAGTATGGGATGATGAATG |  |  |
|  | **INRA121** | GGAAACCCATTGGAGGATTTG | FAM | 106–110 bp |
|  |  | CTTCACTATTCCCCACAAAG |  |  |
|  | **SRCRSP11** | GTGCCCCATCACACATG | PET | 112–132 bp |
|  |  | GTGGTTCTTTACGCTGAGCC |  |  |
|  | **TGLA53** | GCTTTCAGAAATAGTTTGCATTCA | VIC | 132–156 bp |
|  |  | ATCTTCACATGATATTACAGCAGA |  |  |
|  | **SY259** | GCACCACAACAAAGAGGAGC | FAM | 150–166 bp |
|  |  | TGAAGACATAAGGGCGAACAG |  |  |
| **SET3A** | **BM1258** | AAGCCGCGCTTGAAGGAG | VIC | 101–129 bp |
|  |  | ATCAAGCTCCCCTCTTCG |  |  |
|  | **SRCRSP09** | GCAGGACTCTACGGGCCTTGC | NED | 118–136 bp |
|  |  | CACGGAGTCACAAAGAGTCAGACC |  |  |
|  | **ILSTS030** | CTGCAGTTCTGCATATGTGG | PET | 152–180 bp |
|  |  | CTTAGACAACAGGGTTTGG |  |  |
| **SET3B** | **ETH225** | GATCACCTTGCCACTATTTCCT | FAM | 136–156 bp |
|  |  | ACATGACAGCCAGCTGCTACT |  |  |
|  | **SRCRSP06** | CATAGTTCATTCACAATATGGCA | PET | 140–154 bp |
|  |  | CATGGAGTCACAAAGAGTTGAA |  |  |
|  | **BOBT24** | GAGCAAGGGAATTCAGTGGAGC | VIC | 148–176 bp |
|  |  | TGTATTTTACATTCAGGTCTGTGATCC |  |  |
|  | **NRAMP1** | GTGGAATGAGTGGGCACAGT | VIC | 194–220 bp |
|  |  | CTCTCCGTCTTGCTGTGCAT |  |  |
| **SET4** | **MAF214** | GGGTGATCTTAGGGAGGTTTTGGAGGAAT | PET | 215–260 bp |
|  |  | GCAGGAGATCTGAGGCAGGGAC |  |  |

**Table 4:** Microsatellite loci analyzed in fragmentation analysis of *C. capreolus*

| PCR multiplex | Used primer pairs | Sequence5 '– 3' | Fluorescent labeling | Fragment length |
| --- | --- | --- | --- | --- |
|  | **BM1818** | AGCTGGGAATATAACCAAAGG | PET | 247–263 bp |
|  |  | AGTGCTTTCAAGGTCCATGC |  |  |
|  | **BM757** | TGGAAACAATGTAAACCTGGG | NED | 158–211 bp |
|  |  | TTGAGCCACCAAGGAACC |  |  |
| **SET1** | **CSSM66** | ACACAAATCCTTTCTGCCAGCTGA | FAM | 167–185 bp |
|  |  | AATTTAATGCACTGAGGAGCTTGG |  |  |
|  | **NVHRT73** | CTTGCCCATTTAGTGTTTTCT | NED | 210–267 bp |
|  |  | TGCGTGTCATTGAATAGGAG |  |  |
| **SET2** | **NVHRT24** | TGTGGACTATAGGGAGC | FAM | 97–125 bp |
|  |  | GTGTACAAAAAGTGATTGAGT |  |  |
|  | **NVHRT48** | CGTGAATCTTAACCAGGTCT | PET | 80–95 bp |
|  |  | GGTCAGCTTCATTTAGAAAC |  |  |
|  | **NVHRT16** | ATTCTAAGCCCAAATAATCTT | NED | 151–175 bp |
|  |  | TCTAAGGGGTCTGTGTCTT |  |  |
|  | **RT1** | TGCCTTCTTTCATCCAACAA | FAM | 210–245 bp |
|  |  | CATCTTCCCATCCTCTTTAC |  |  |
| **SET 3** | **MCM64** | TACAGTCCATGGGGTCACAAGAG | PET | 123–153 bp |
|  |  | TCTGAATCTACTCCCTCCTCAGAGC |  |  |
|  | **Roe1** | AAATTTGGCTCTGCAATCGG | PET | 131–133 bp |
|  |  | ACACAAAAGCCACCCAATAC |  |  |
|  | **ETH225** | GATCACCTTGCCACTATTTCCT | NED | 137–155 bp |
|  |  | ACATGACAGCCAGCTGCTACT |  |  |
|  | **ROE8*** | AAGCCGCGCTTGAAGGAG | FAM | 59–101 bp |
|  |  | ATCAAGCTCCCCTCTTCG |  |  |
| **SET 4** | **MAF70** | GCAGGACTCTACGGGCCTTGC | PET | 117–155 bp |
|  |  | CACGGAGTCACAAAGAGTCAGACC |  |  |
|  | **NVHRT21** | GCAGCGGAGAGGAACAAAAG | PET | 157–178 bp |
|  |  | GGGGAGGAGCAGGGAAATC |  |  |
