# Supplementary material for: Genetic characterisation of wild ungulates: successful isolation and analysis of DNA from widely available bones can be cheap, fast and easy
Source: Zookeys. 2020 Sep 3;965:141–56. doi: 10.3897/zookeys.965.54862 (PMC7483325; doi:10.3897/zookeys.965.54862)

**Supplementary Material 2**

**Figure 1.** Microsatellite loci for muscle tissue samples (upper) and recent bone samples (bottom) of *R. rupicapra* isolated using the cost-efficient PeqLab kit. Mulitilocus genotypes from SET1, SET2 and SET3 are presented (see Supplementary Material 1).


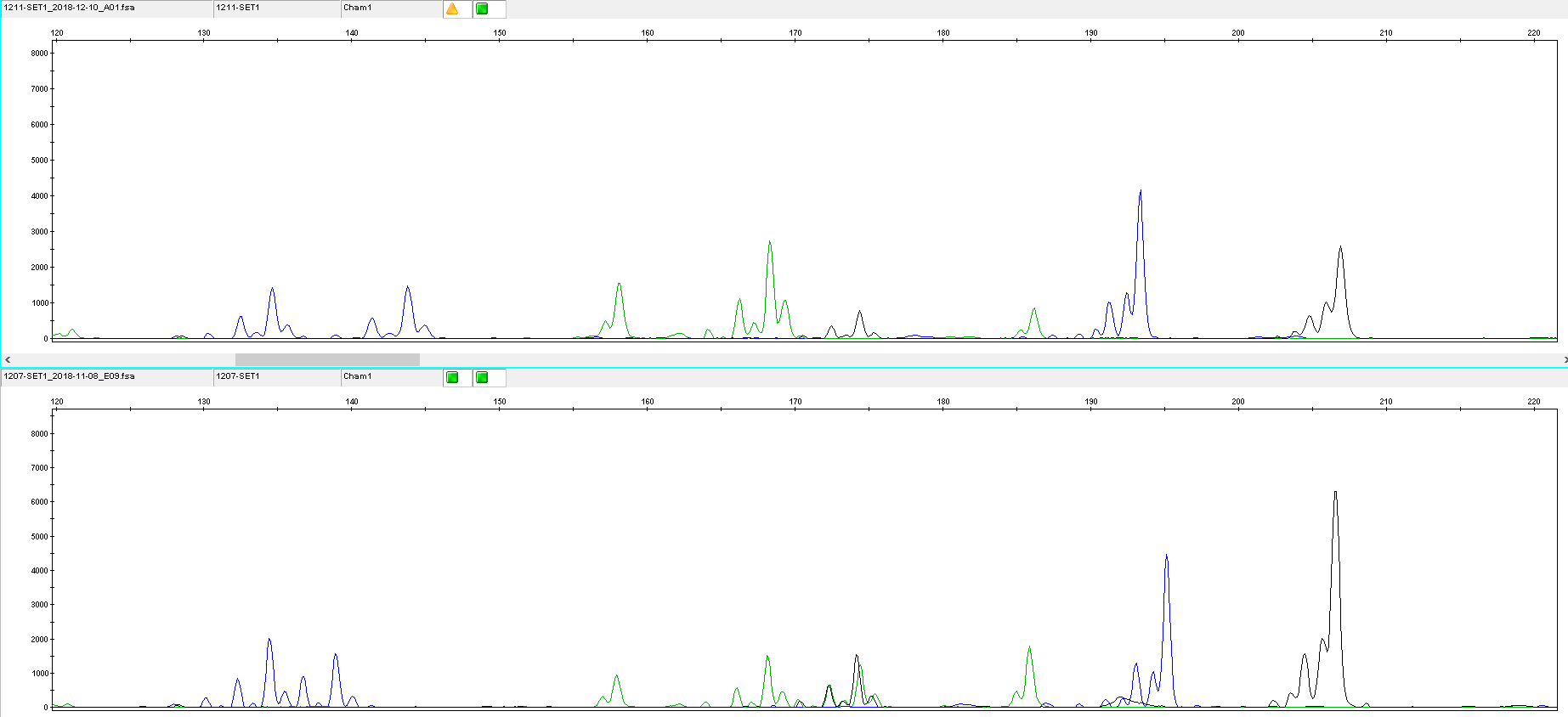


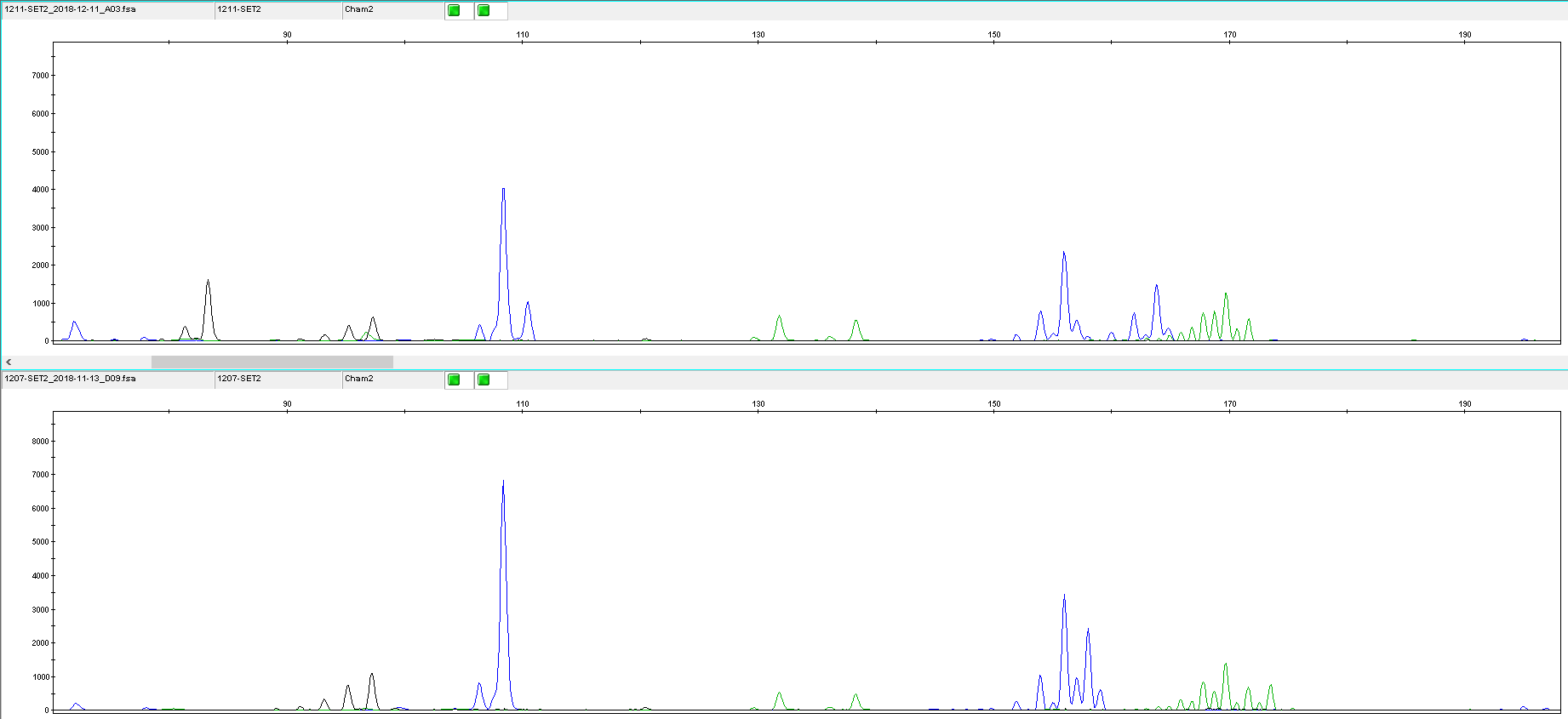


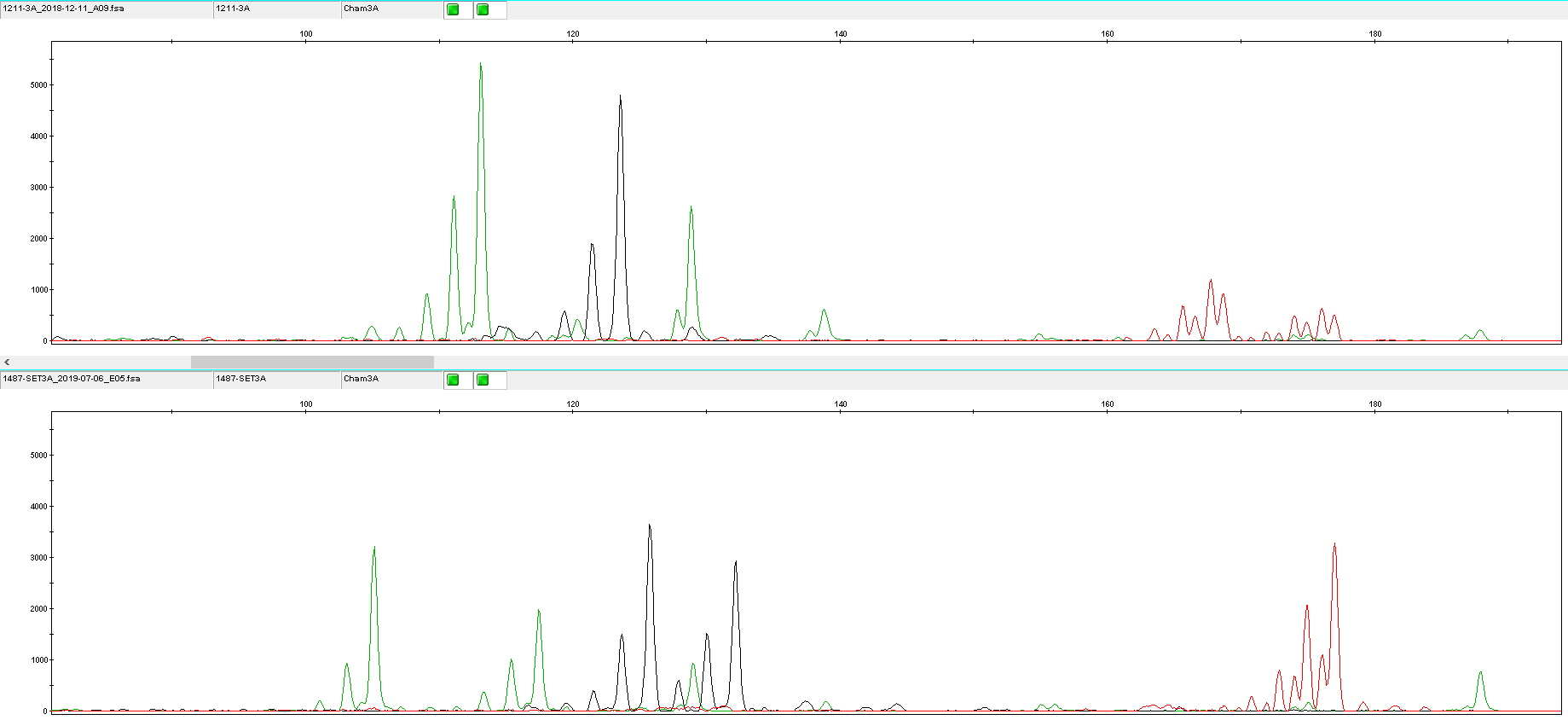


**Figure 2.** Microsatellite loci for muscle tissue samples (bottom) and recent bone samples (upper) of *C. capreolus* isolated with cost efficient PeqLab kit. Mulitilocus genotypes from SET1, SET2, SET3 and SET4 are presented (see Supplementary Material 1).


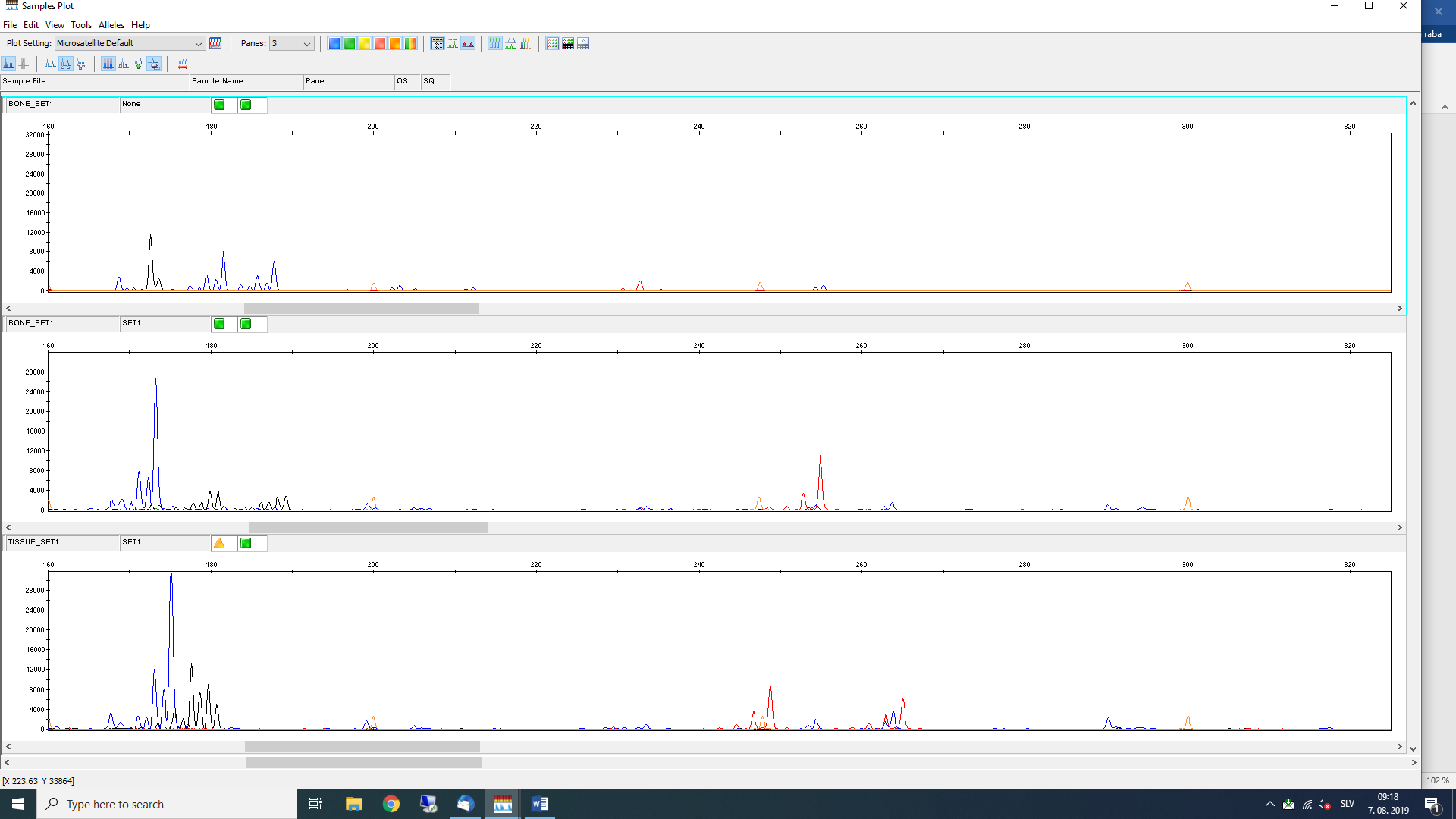


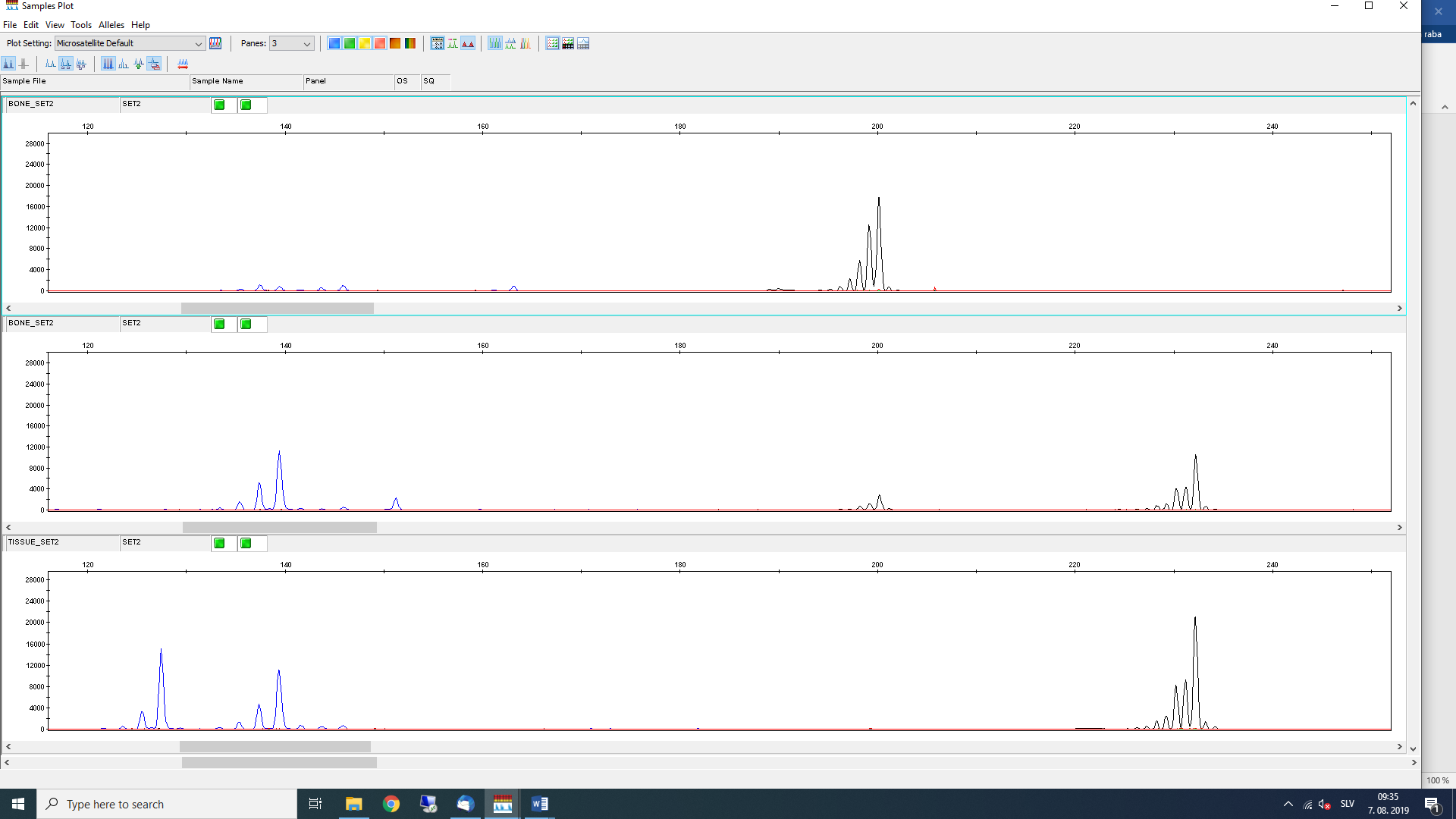


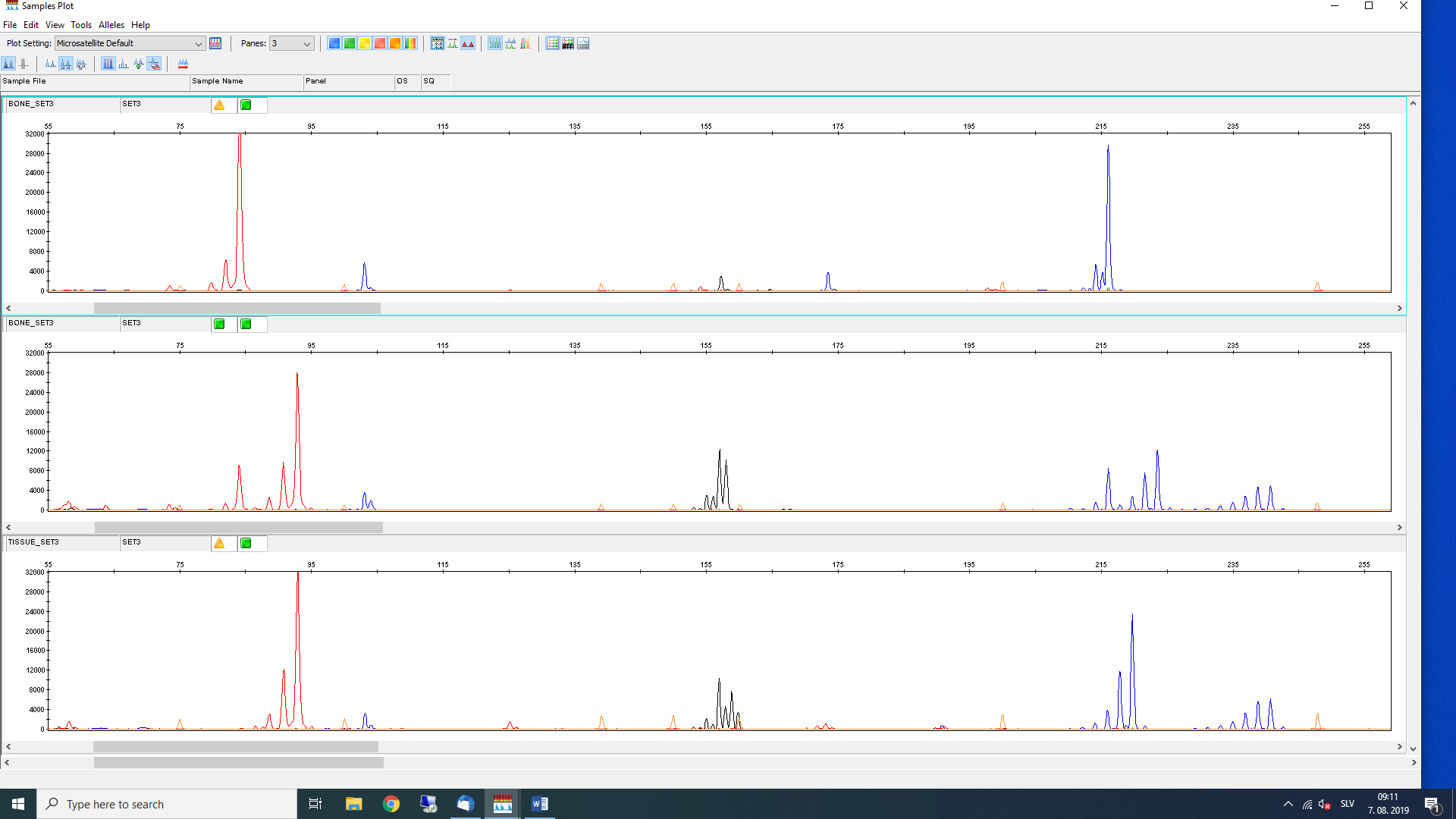


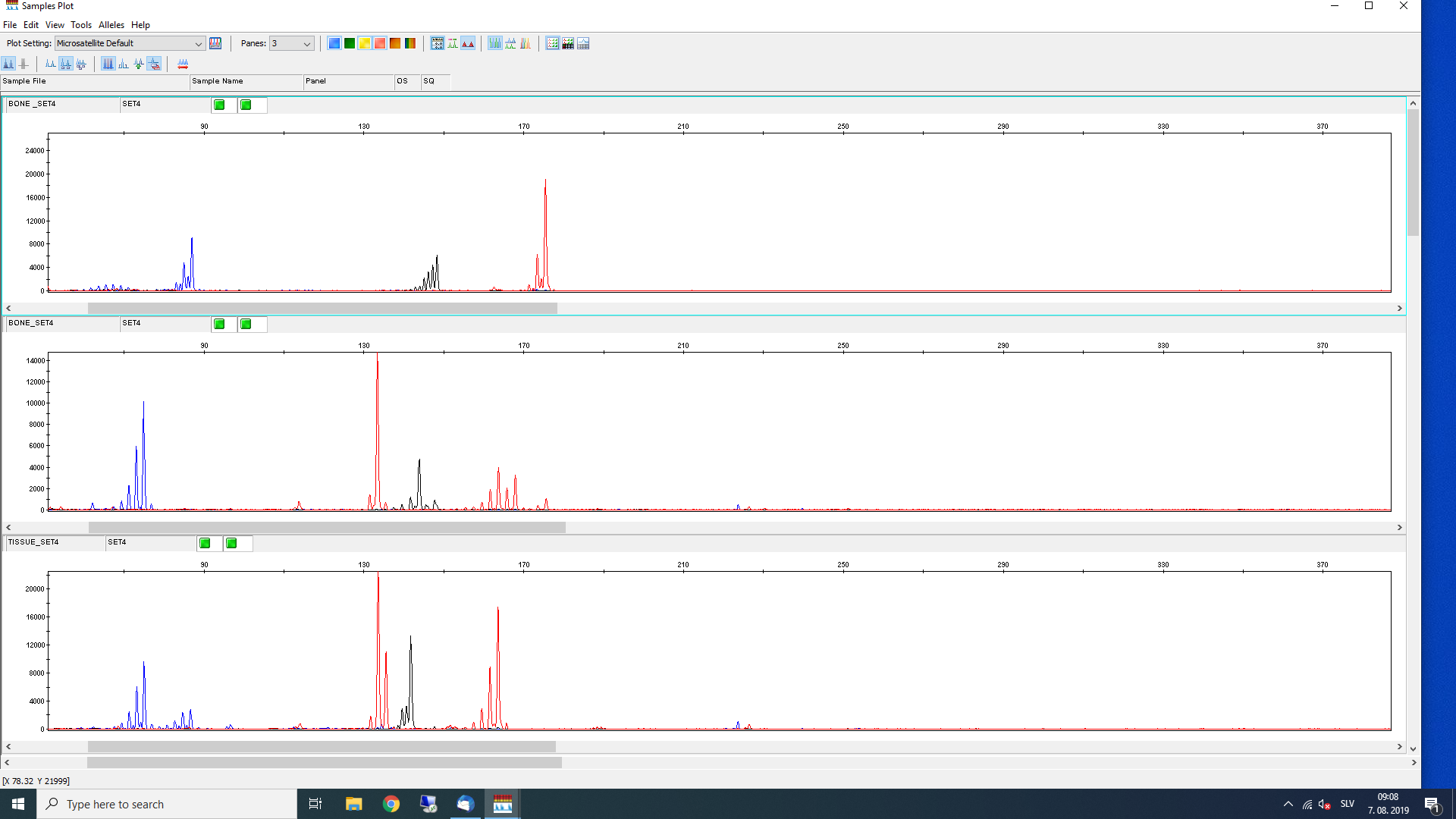

Supplement: Supplementary material 2 — Microsatellite loci for muscle tissue samples and recent bone samples [file zookeys-965-141-s002.doc]
